# Pour some sugar on me: Glycemic control and the prevention of liver disease

**DOI:** 10.1097/HC9.0000000000000400

**Published:** 2024-03-04

**Authors:** Vincent L. Chen, Jennifer A. Flemming

**Affiliations:** 1 Department of Internal Medicine, Division of Gastroenterology and Hepatology, University of Michigan Medical School, Ann Arbor, Michigan, USA; 2 Departments of Medicine and Public Health Sciences, Queen’s University, Kingston, Ontario, Canada

There has been substantial growth in the prevalence of type 2 diabetes mellitus worldwide, which has paralleled an increase in the burden of chronic liver diseases and HCC. These trends may be interlinked. Diabetes is associated with both significant liver fibrosis and fibrosis progression.^1^ Associations between diabetes and the development of cirrhosis and HCC have also been well documented, including 2 studies from a nationwide US Veterans Affairs (VA) cohort and a Hong Kong population-based cohort found that in people with diabetes (most of whom did not have known baseline cirrhosis), adequate glycemic control was associated with lower incidence of HCC.^2,3^ Another territory-wide study from Hong Kong found that among people with diabetes, with or without documented chronic liver disease, a longer duration of disease was associated with a higher risk of liver-related events, including HCC.^4^ Among patients with chronic hepatitis B followed in China, a longer duration of diabetes and higher hemoglobin A1c were associated with a higher risk of HCC.^5^ These data suggest that the optimization of glycemic control among those with diabetes could translate into improved liver-related outcomes, which would have important clinical implications.

However, there are notable limitations in the literature. There are minimal data on the impact of diabetes and glycemic control on HCC in people with underlying cirrhosis. Accordingly, it remains unclear if the association between glycemic control and HCC in the overall population is driven by an increased risk of fibrogenesis or independent oncogenic processes. Most of the studies investigating the association between diabetes and HCC risk in populations without cirrhosis were in Asian countries or hospital-based cohorts, and their applicability to the general Western population is less clear. In addition, it is unclear if the risk of cirrhosis and HCC are higher among the population with prediabetes (defined as abnormal glucose levels not reaching the definition of diabetes mellitus), which would further strengthen the argument of the importance of glycemic control. In this edition of *Hepatology Communications*, 2 studies address some of these limitations and further our understanding of this complex relationship.

In the first study by Ye et al, the authors use the UK BioBank (UKBB) to perform a retrospective cohort study in an attempt to further define these associations in the general population aged 40–69 years.^6^ They show that the risk of cirrhosis and HCC are associated with having prediabetes and estimated future risk of diabetes (defined using FINnish Diabetes RIsk SCore [FINDRISC] quintiles^7^), worse glycemic control (baseline hemoglobin A1C >7.5%), and a longer duration of diabetes (>5 y). The authors also found that the FINDRISC score interacted with the genetic risk of HCC, so that in those predisposed to HCC based on their genetics, the effect of high FINDRISC was even greater than in those at lower genetic HCC risk.

In the second study, Mezzacappa et al studied a cohort of 81,907 Veterans with cirrhosis.^8^ They found that prevalent diabetes was associated with a modestly increased incidence of HCC (adjusted subhazard ratio [asHR] 1.18). There was heterogeneity in HCC risk across disease etiology, most notably with no significant association between diabetes and HCC incidence in patients with chronic hepatitis C (HCV) related cirrhosis. The authors then divided patients based on glycemic control, with sustained blood glucose control, nonsustained glucose control, and uncontrolled glucose as hemoglobin A1c < 7% for ≥2 years, 0–2 years, and not at all, respectively. Nonsustained glucose control and uncontrolled glucose were associated with a higher incidence of HCC compared to sustained glucose control across the Child-Pugh class, with an asHR of 1.8–2.4. This study noted a few surprising findings as well. First, a more recent diagnosis of diabetes relative to the date of cirrhosis diagnosis was associated with a higher risk of HCC, with no association between prevalent diabetes and incident HCC in those with diabetes 5–10 years before the cirrhosis diagnosis. Second, patients taking insulin use had a lower incidence of HCC than those who did not.

Together, these studies suggest that management of underlying diabetes and specifically glycemic control may impact the development of cirrhosis and HCC, reinforcing the importance of a multidisciplinary approach to combat the development of liver disease and liver cancer in those with diabetes. That these 2 studies yielded similar results in 2 very different patient populations—the UK Biobank, which is community-based and typically “healthier” than the general population, and a US cohort of patients with baseline cirrhosis and a higher burden of comorbidities than the general population—strengthens the findings.

These studies are however not without limitations. First is the lack of accounting for the use of medications. Multiple cohort studies have suggested that both metformin and statins are associated with reduced risks of cirrhosis and HCC,^9^ yet the VA study did not account for metformin use, and the UK Biobank study did not account for either. Other time-varying co-variates that can impact the development of cirrhosis and HCC (changes in body mass index, alcohol consumption etc.) were also not accounted for. Given the long follow-up time in the UK Biobank cohort (median 12 y), time-varying exposures are also of importance, especially the development of incident diabetes during the follow-up period. This is particularly important for the analysis related to prediabetes using the FINDRISC quintiles, given that the median FINDRISC score of the cohort was 8, which is associated with a 1%–17% risk of incident diabetes over 10 years.^7^ Without using a time-varying approach in this subset, it may be that those in the higher FINDRISC quintiles developed diabetes during follow-up, driving the observed association with liver events. Finally, in the UK Biobank study, it was also unclear if co-morbid HCV was adequality excluded in the baseline population. In the cohort flow chart, only n = 269 individuals with HCV were identified, giving a prevalence of 0.05%, ~ 10-fold lower than the documented prevalence in the UK, and less prevalent than HBV, autoimmune liver diseases, and hereditary liver disease, which would be inconsistent with known epidemiology. As HCV has been associated with the development of incident diabetes,^10^ there is concern that a proportion of individuals identified with diabetes may also have co-morbid HCV, which could confound the observed associations. This is also important considering the results of the VA study, where the association between diabetes and HCC was not observed among those with HCV cirrhosis.

One notable difference in the results between the 2 studies was the association between the duration of diabetes and the risk of HCC. If the theory that worse glycemic control is promoting the development of HCC is correct, one would expect that the longer the duration of diabetes, the higher the risk of HCC. Indeed, in the UK Biobank study, those with a diagnosis of diabetes for >5 years had the highest hazard for HCC (asHR 9.34) compared to <5 years (asHR 5.79) or those with prediabetes (asHR 2.25). Conversely, in the study from the VA, the risk of HCC was highest among those with the shortest duration of diabetes (0–2 y) relative to their diagnosis of cirrhosis and lowest in those with a duration of diabetes of 10+ years. These differences could relate to differences in the underlying populations studied (general population vs. population with cirrhosis). Another possibility is that in the VA study, those with a longer duration of diabetes had improved glycemic control as they were under the care of diabetes practitioners and exposed to medications that have been shown to be associated with a reduction in HCC (ie, metformin/statins). As the use of these medications was not evaluated in either study, future studies that can account for medication use may help reconcile the difference in results. In addition, diagnosis codes for diabetes and cirrhosis and its complications have been validated within the VA cohort,^11^ but not to our knowledge in UKBB.

These studies add to the literature which support the concept that optimization of glycemic control in those with diabetes is an important part of multidisciplinary management to improve liver-related outcomes. These data have important implications for clinical care. To verify these findings, future prospective studies are needed. Cohorts of patients with cirrhosis followed to identify the risk of HCC and/or hepatic decompensation associated with diabetes should stratified by glycemic control and account for the dose and duration of key medications.
